# Supplementary material for: University student wellbeing during COVID-19: associations with infection prevalence and social gathering restrictions in an observational study
Source: Front Psychiatry. 2026 Jan 14;16:1641305. doi: 10.3389/fpsyt.2025.1641305 (PMC12848539; doi:10.3389/fpsyt.2025.1641305)
Supplement: Supplementary file 1 [file Table1.docx]

Supplementary Material

# Table S1. Class Demographics, University COVID-19 Cases, and Percent At-Risk Values

|  | **F20** | **F21** | **S22** | **S23** | **S24** | **AVG/TOT**^a^  (% of full data set) |
| --- | --- | --- | --- | --- | --- | --- |
| **Class Composition** |  |  |  |  |  |  |
| Freshman (% of class) | 3 (6.4) | 4 (6.2) | 13 (18.8) | 1 (2.1) | 0 (0) | 21 (7.6) |
| Sophomore (% of class) | 17 (36.2) | 19 (29.2) | 11 (15.9) | 14 (29.8) | 12 (24.5) | 73 (26.4) |
| Junior (% of class) | 15 (31.9) | 22 (33.8) | 10 (14.5) | 10 (21.3) | 20 (40.8) | 77 (27.8) |
| Senior (% of class) | 12 (25.5) | 20 (30.8) | 35^b^ (50.7) | 22 (46.8) | 17 (34.7) | 106 (38.3) |
| Total Enrolled (% of full data set) | 47 (17.0) | 65 (23.5) | 69^b^ (24.9) | 47 (17.0) | 49 (17.7) | 277 |
| **Ethnicity and Legal Sex^c^** |  |  |  |  |  |  |
| Asian |  |  |  |  |  | 26 (22.6) |
| Black |  |  |  |  |  | 22 (19.1) |
| Hispanic |  |  |  |  |  | 17 (14.8) |
| Multi |  |  |  |  |  | 2 (1.7) |
| Unknown |  |  |  |  |  | 2 (1.7) |
| White |  |  |  |  |  | 42 (36.5) |
| International |  |  |  |  |  | 4 (3.5) |
| Female |  |  |  |  |  | 85 (73.9) |
| **Local COVID-19 Cases** |  |  |  |  |  |  |
| Dekalb County (% of full data set) | 10,179 (16.8) | 16,603 (27.4) | 28,368 (46.8) | 5,426^e^ (9.0) | No data | 60,576 |
| Emory Total^d^ (% of full data set) | 211 (10.0) | 606 (28.6) | 976 (46.1) | 323 (15.3) | No data | 2,116 |
| Emory Students (% of full data set) | 149 (10.7) | 466 (33.6) | 605 (43.6) | 168 (12.1) | No data | 1,388 |
| **% At-risk for Poor Wellbeing** |  |  |  |  |  |  |
| GAD-7 (mean ± SD) | 25.99 ± 6.40 | 39.04 ± 5.78 | 35.78 ± 7.44 | 34.58 ± 7.52 | 30.83 ± 7.87 | 33.24 |
| PHQ-2 (mean ± SD) | 29.62 ± 5.52 | 34.47 ± 4.02 | 26.52 ± 7.72 | 21.75 ± 7.11 | 21.05 ± 5.02 | 26.68 |
| WHO5 (mean ± SD) | 65.28 ± 9.16 | 63.34 ± 6.72 | 62.52 ± 9.11 | 55.89 ± 9.66 | 45.01 ± 7.70 | 58.41 |

**^a^*AVG/TOT = average and total***

***^b^2 Seniors included in total withdrew***

***^c^Ethnicity and Legal Sex is presented in aggregate form and does not include all students due to incomplete university datasets***

***^d^Includes students, faculty, and staff***

***^e^County case numbers were not reported the last three weeks of S23***

# Table S2. Survey Instrument^a^

| **GAD-7:** “Over the **last week**, how often have you been bothered by the following problems?”  *0 — Not at all; 1 — Several days;*  *2 — More than half the days; 3 — Nearly every day*  **Q1. Feeling nervous, anxious, or on edge**  **Q2. Not being able to stop or control worrying**  **Q3. Worrying too much about different things**  **Q4. Trouble relaxing**  **Q5. Being so restless that it's hard to sit still**  **Q6. Becoming easily annoyed or irritable**  **Q7. Feeling afraid as if something awful might happen**  *Recommended Cutoff:* **10 or greater** |
| --- |
| **PHQ-2**: “Over the **last week**, how often have you been bothered by the following problems?”  *0 — Not at all; 1 — Several days;*  *2 — More than half the days; 3 — Nearly every day*  **Q1. Little interest or pleasure in doing things**  **Q2. Feeling down, depressed, or hopeless**  *Recommended Cutoff:* **3 or greater** |
| **WHO5:** “Please indicate how you have been feeling over the **last week**for each of the five statements.”  *0 — At no time; 1 — Some of the time; 2 — Less than half of the time;*  *3 — More than half of the time; 4 — Most of the time; 5 — All of the time*  **Q1. I have felt cheerful and in good spirits**  **Q2. I have felt calm and relaxed**  **Q3. I have felt active and vigorous**  **Q4. I woke up feeling fresh and rested**  **Q5. My daily life has been filled with things that interest me**  *Recommended Cutoff:* **below 13** |

**^a^*Surveys were administered in the Canvas Learning Management System;***

***students used dropdown menus to select scores for each question.***

# Results S1. Scale Means

Overall Descriptive Outcomes: Across cohorts, the mean (SD) total scores for each scale were 7.8 ± 1.0 (GAD-7, out of 21), 1.8 ± 0.3 (PHQ-2, out of 6) and 11.5 ± 1.2 (WHO5, out of 25). Anxiety, depression, and subjective wellbeing all indicated lowest wellness during the F21 semester, with average scores of 8.7 ± 0.8 (GAD-7), 2.2 ± 0.2 (PHQ-2) and 10.9 ± 0.6 (WHO5). By S24, scores improved to 7.2 ± 0.9 (GAD-7), 1.5 ± 0.2 (PHQ-2) and 12.9 ± 0.7 (WHO5). For all three scales, one-way ANOVA revealed a significant effect of semester (GAD-7: *F*_(4, 64)_ = 10.3, *P* < .0001; PHQ-2: *F*_(4, 64)_ = 15.8, *P* < .0001; WHO5: *F*_(4, 64)_ = 14.7, *P* < .0001). To further examine differences between semesters while minimizing Type I error, we applied Tukey's multiple comparisons tests for each scale.

GAD-7: Anxiety levels rose significantly from F20 to F21 (difference, -1.6; 95% CI, -2.41 to -0.72; *P* < .0001) and S22 (difference, -1.2; 95% CI, -2.02 to -0.33; *P* = .0020), whereas they did not differ between F20 and the later semesters (S23/24). Anxiety scores decreased from F21 to S23 (difference, 0.97; 95% CI, 0.14 to 1.79; *P* = .0137) and further decreased in S24 to drop below F21 (difference, 1.4; 95% CI, 0.62 to 2.27; *P* < .0001) and S22 (difference, 1.1; 95% CI, 0.23 to 1.88; *P* = .0058) levels.

PHQ-2: Depression levels rose significantly from F20 to F21 (difference, -0.31; 95% CI, -0.58 to -0.036; *P* = .0188) and then dropped from F21 to S22 (difference, 0.41; 95% CI, 0.14 to 0.68; *P* = .0007) such that there was no difference between F20 and S22. Depression scores fell further in S23 from F20 (difference, 0.30; 95% CI, 0.021 to 0.57; *P* = .0283) and F21 (difference, 0.61; 95% CI, 0.34 to 0.88; *P* < .0001) and were significantly decreased in S24 from F20 (difference, 0.37; 95% CI, 0.10 to 0.65; *P* = .0026), F21 (difference, 0.68; 95% CI, 0.42 to 0.95; *P* < .0001), and S22 (difference, 0.28; 95% CI, 0.0093 to 0.55; *P* = .0391) levels.

WHO5: There were no differences in average WHO5-assessed wellbeing between the first four semesters. In contrast, scores increased significantly in S24 compared to all prior semesters (F20: difference, -2.0; 95% CI, -2.97 to -1.10; *P* < .0001; F21: difference, -2.1; 95% CI, -3.00 to -1.15; *P* < .0001; S22: difference, -2.0; 95% CI, -2.93 to -1.09; *P* < .0001; and S23: difference, -1.2; 95% CI, -2.15 to -0.31; *P* = .0033).
